# Balance between Smog Control and Economic Growth in China: Mechanism Analysis Based on the Effect of Green Technology Innovation

**DOI:** 10.3390/ijerph20021475

**Published:** 2023-01-13

**Authors:** Kai Yuan, Yabing Qin, Chenlu Wang, Zihao Li, Tingting Bai

**Affiliations:** 1 Department of Economics and Management, Bozhou University, Bozhou 236800, China; 2 School of International Business and Economics, Henan University of Economics and Law, Zhengzhou 450046, China; 3 School of Business, Nanjing University of Information Science and Technology, Nanjing 210044, China; 4 Yangtze Institute for International Digital Trade Innovation and Development, Nanjing 210044, China; 5 School of Business Administration, Northeastern University, Shenyang 110189, China

**Keywords:** green technology innovation, smog pollution, economic growth, mechanism analysis

## Abstract

The balance between smog pollution (SP) control and economic growth (EG) is currently a major problem facing China’s development. Green technology innovation (GTI) is an effective way to promote ecological civilization and realize green development. Thus, whether GTI can facilitate a win–win situation of SP control and stable EG is an important issue of academic concerns. In this paper, the mechanisms of the role of GTI, SP and EG were systematically demonstrated. The corresponding research hypotheses were proposed. Based on the data book of 278 Chinese cities from 2008 to 2020, the effects of GTI on SP and EG were systematically investigated using the econometric estimation method of dynamic spatial panel simultaneous equations. The results show that GTI can reduce SP directly, or indirectly by promoting EG. Although GTI can promote EG, EG may be inhibited due to GTI-induced SP reduction. Inter-regional SP showed significant spatial agglomeration characteristics. EG had significant spatial correlation effects. GTI in neighboring regions can also facilitate local SP control. Further analysis shows that compared with green utility model innovation (GUMI), green invention and innovation (GII) had a more significant effect on reducing SP and promoting EG. In addition, the analysis of the comprehensive effect of GTI on SP and EG shows that GTI can achieve the overall balanced development of SP prevention and EG regardless of GTI types.

## 1. Introduction

The report of the 19th National Congress of the Communist Party of China (CPC) points out that development is the basis and key to solving all problems in China and that innovative, coordinated, green, open and shared development must be unswervingly implemented. Particularly, innovation and green innovations are highlighted. Regarding the practical challenges of China’s green development, smog pollution (SP) is particularly serious. In December 2016, China’s persistent large-scale smog affected 17 provinces, autonomous regions and municipalities, covering an area of 1.42 million km^2^. The population in heavily polluted areas reached 260 million. In 2018, only 33.7% of the 338 monitored cities met air quality standards. Resolutely winning the battle for a blue sky is the top priority, which Comrade Xi Jinping stressed at the National Conference on Ecological and Environmental Protection. Since the 18th Party Congress, the CPC Central Committee and governments at all levels have attached great importance to smog management. They introduced a series of smog management policies and measures and invested many resources, with certain achievements. However, smog management in most areas still depends on mandatory adjustment, total compression and campaign-style control. Although this mode has caused short-term smog relief, the downward economic pressure brought by smog management cannot be ignored. It has been predicted that the smog management policies for air pollution prevention and the measures for enhancing air pollution prevention and control in Beijing, Tianjin and Hebei would result in a total loss of 631.5 billion yuan (8.45% of the total regional GDP) and 145.95 billion yuan (16.05%) of GDP in 2017 and 2020, respectively (from the “Beijing-Tianjin-Hebei Haze Control Policy Assessment Report” completed by Professor Shi Minjun in 2017).

Currently, the downward economic pressure is increasing in China due to some factors such as the China–United States trade war and the COVID-19 pandemic. Thus, the pollution control battle in China will continue to deepen. How to resolve the contradiction between SP control and economic growth (EG) and achieve a win–win situation of SP reduction and stable EG? Green technology innovation (GTI) is a key emerging technology for the harmonious coexistence of humans and nature. It is also an important emerging field in the new global industrial revolution and technological competition. GTI has become an important pathway to tackle the contradiction and achieve the win–win situation for environmental improvement and EG [1]. However, many problems still exist in China’s GTI, such as insufficient kinetic energy and underdeveloped GTI standards and policies (Ministry of Science and Technology, 2019). China’s relatively extensive EG mode of resource and energy consumption needs to be continuously optimized and transformed. Thus, can GTI in various regions achieve the win–win situation? Currently, China is in the critical stage of building an innovative country and the pollution control battle. Its stable macroeconomic growth is faced with unprecedented challenges. Therefore, studying how GTI can effectively achieve the win–win situation in the new era of socialism is significant to promote high-quality EG and ecology-prioritized green development strategies.

The marginal contribution is mainly reflected in the following three aspects: firstly, the mechanism. The influencing mechanism of GTI was constructed to balance regional EG and SP. The two-way effects of GTI were introduced through the interactive effects of SP and EG. The direct and indirect influencing mechanisms of GTI on SP control and EG were constructed. Thus, the complex effects of GTI on SP and EG can be demonstrated more objectively and comprehensively. Secondly, research methods can be utilized. A more systematic and scientific spatial panel econometric equation estimation method was used. In this paper, the interaction between SP control and EG was controlled by constructing a spatial panel simultaneous equation. The spatial interactions between regional GTI, SP and EG were also considered. This allows for a more scientific study of related mechanisms. Thirdly, research levels can be accounted for. Long time series data at the provincial level and microdata at the municipal level were comprehensively used. Using the long time series data of 30 provinces from 1998 to 2020, we observed and analyzed the objective relationship between GTI, SP and EG over a long period. The effects of GTI were investigated at a more microscopic level by manually collecting and sorting relevant data of 216 Chinese cities during 2008–2020.

This paper is arranged as follows: following the introduction, Section 2 presents the literature review; Section 3 presents the theoretical mechanisms and research hypotheses; Section 4 presents the causality and spatial relationship test; Section 5 presents the study and design; Section 6 presents the empirical analysis results and discussion; Section 7 presents conclusions and implications.

## 2. Literature Review

The classic study by Grossman and Krueger found an inverted U-shaped environmental Kuznets Curve (EKC) between EG and air pollution, highlighting the relationship between SP and EG [2]. Relevant studies can be grouped into two categories. (1) The impact of EG on SP. Based on the EKC framework, it is found that SP became a by-product of China’s rapid EG due to the constraints of extensive EG [3]. A study [4] on Asian countries found that EG exacerbated atmospheric SP. Zhu et al. (2019) and Ma et al. (2019) further examined the impact of EG on SP in China and found that the decoupling stage of SP and EG at the regional level has not occurred yet based on the EKC [3,5]. It is also believed that EG can reduce SP [5,6]. For example, Aslanidis and Iranzo’s study of gaseous pollutants in 77 non-OECD countries found that when EG reached a certain level, factors such as increased public demand for environmental quality and transformation of EG modes could lead EG to improve SP [7,8]. (2) The impact of SP on EG. Zhang et al. (2022) argued that environmental pollution could pressure sustained and stable EG by changing people’s preferences for environmental quality and adjusting the industrial structure [9]. However, it is also found that SP can significantly reduce the EG quality in China through two important pathways: impeding urbanization and reducing human capital accumulation [10]. In addition, mandatory government policies during SP control, such as pollution taxes and energy control, can also negatively impact EG [11].

GTI is a general term for processes and technologies that mitigate environmental pollution, reduce resource and energy consumption and improve the ecological environment. (1) The impact of GTI on EG. Su et al. (2022) believed that technological progress was a driving force in promoting EG [12], and GTI could make low-input–high-output intensive production the mainstay of EG. This can help to avoid the environmental pollution caused by traditional technology innovation in promoting EG [10]; namely, clean technology innovation can achieve sustainable EG [13]. (2) The impact of GTI on SP. Firstly, regarding the inhibitory effect of GTI on pollution, the Porter hypothesis suggests that strict environmental regulations can promote GTI to achieve SP control [14]. Pollution emissions are mainly reduced by transforming traditional production technologies and improving productivity using energy-saving and emission-reducing technologies [15]. In addition, GTI has spatial agglomeration characteristics, and regional GTI shows imitation behavior among neighboring regions. These can synergistically improve inter-regional GTI efficiency and environmental resource benefits [16]. Secondly, it is found that increased GTI levels can directly reduce local and neighboring SP through the spatial spillover effect of GTI [17].

The relevant studies have laid a solid foundation for subsequent research, with some limitations. Studies on the relationship between SP and EG mainly include investigations of directional effects, while studies on interactive effects are relatively lacking. The anti-smog effect of GTI is rarely involved. There is a lack of sound empirical studies on achieving a win–win situation of SP and EG through GTI. Therefore, this paper attempts to address these existing research issues.

## 3. Theoretical Mechanisms and Research Hypotheses

A causal relationship exists between EG and SP. Firstly, during rapid EG, economic activity factors such as accelerated industrialization and increased energy consumption by urbanization can exacerbate SP [6,18]. Then, increased EG levels can gradually increase public awareness of environmental protection. This can also increase the pressure on local governments to protect the environment [9]. As a result, SP problems can be mitigated. Secondly, SP is an undesirable output during extensive EG [5,6]. Factors causing SP, such as energy consumption, industrialization and infrastructure construction, are important driving forces of EG. Under the development mode dominated by extensive EG, intensifying SP will promote regional EG. However, SP may reduce the EG quality [10]. In addition, government investment in SP control can also have a crowding-out effect on enterprise performance [19], adversely affecting EG.

### 3.1. Analysis of the Impact of GTI on SP

The impact of GTI on SP includes two aspects. (1) Direct impact of GTI on SP. GTI can directly reduce SP, mainly because GTI can reduce the energy use intensity for transforming and upgrading traditional technologies. Developing new products and technologies during GTI can also reduce energy consumption and pollution emissions [15], facilitating SP control. In addition, the imitation behavior of neighboring GTI [17] and GTI’s spatial spillover effects on SP control [6] can promote inter-regional coordinated SP control. (2) Indirect impact of GTI on SP by promoting EG. In the initial stage, GTI can promote the transformation of EG modes to intensive modes, thus achieving the transformation and upgrading of industrial structure and green EG [16]. This is conducive to reducing regional SP in the long term. GTI can promote regional EG and thus provide more financial support for clean technology research and development (R&D) and innovation in economically developed regions. Then, pollutant emissions can be reduced [20,21]. Higher EG levels can further positively promote clean technology R&D and enterprise investment, which is conducive to inhibiting regional SP. The public’s demand for quality of life and environmental protection increases due to higher GTI-induced EG. This can motivate local governments to adopt higher environmental regulatory standards [15], facilitating SP control.

**Hypothesis 1.**  *Increasing GTI can reduce SP directly, or indirectly by boosting EG*.

### 3.2. Analysis of the Impact of GTI on EG

The impact of GTI on EG can also be divided into two aspects. (1) Direct impact of GTI on EG. GTI can promote enterprises’ cleaner production, production efficiency, product quality and total factor productivity, thus promoting EG [12]. Continuous green technology progress can promote regional industrial transformation and upgrades [16]. Thus, modern intensive production modes (e.g., mechanized, specialized and automated modes) can promote EG [10]. In addition, increased R&D investment by GTI, spatial transfer and exchange of GTI and spatial spillover effects of EG can also facilitate inter-regional coordinated EG [22]. (2) Indirect impact of GTI on EG. GTI may indirectly inhibit EG by reducing SP. Firstly, in the current critical stage of economic transformation, most regions in China have not yet achieved fundamental changes in their EG modes. Their development modes and industrial structure and lifestyle adjustment involved in SP control through clean technologies are not conducive to EG in the short term [23]. Therefore, increased GTI input in promoting SP control can reduce the utilization efficiency of traditional production resources, reducing the output at the expense of some EG modes accordingly [19]. In addition, green technologies for SP control can reduce the use of traditional polluting energy sources and increase energy substitution costs. This is detrimental to improving economic efficiency [3] and can even reduce public welfare and employment and increase the downward economic pressure [11]. Finally, increasing GTI levels promote inter-regional environmental standards, thus reducing the introduction of regional polluting industries [16]. This indicates that EG is beneficial to mitigating SP, but regional EG may slow down. Thus, a research hypothesis was proposed:

**Hypothesis 2.**  *Increasing GTI levels can directly promote EG, but indirectly inhibit EG by mitigating regional SP*.

### 3.3. Impact of Different Types of GTI on SP and EG

Patents are usually divided into invention patents, utility model patents and design patents according to the degree of innovation from high to low. Due to the low degree of innovation of design patents, GTI in this paper mainly includes green invention innovation (GII) and green utility model innovation (GUMI). Compared with GUMI, GII represents higher patent quality and technological content, which belongs to value-creating GTI [24]. Based on the differences in the value of GII and GUMI and existing study findings, this paper argues that the impacts of GII and GUMI on regional EG and SP also vary.

(1) Impact of different types of GTI on EG. Firstly, in order to obtain external support, enterprises commonly take GUMI activities to whitewash innovation performance. This can lead to patent bubbles or patent innovation illusions. Such innovation does not focus on patent quality and its practical application values. It may disconnect from the purpose of national EG and the actual demand for enterprise innovation capability improvement. This may distort the relationship between innovation patents and EG [25]. However, high-quality GII has innovation spillover effects and high industrial application values and can effectively promote EG [26]. Secondly, the strategic behavior of pursuing quantity in GUMI activities cannot effectively improve innovation capability, GTI and EG. However, increasing substantive innovation (e.g., invention patents) can enable enterprises to obtain competitive advantages and improve market value [25]. This innovation can also effectively promote enterprise value creation and is an important driving force of EG. (2) Impact of different types of GTI on SP. Firstly, due to the high technological content and patent quality, GII can effectively promote technological progress and green growth and improve environmental quality [27]. This shows that, compared with GUMI, GII induces a higher level of clean technologies, with more significant SP control effects. Secondly, since GII can actively respond to the government’s call for energy-saving and emission-reduction policies, the GUMI level does not change significantly [28]. This indicates that improving the GII level can better facilitate enterprises’ active emission reduction activities, promote green upgrading of industrial structure and production methods and reduce SP. Thus, a research hypothesis was proposed:

**Hypothesis 3.**  *Compared with GUMI, GII may promote EG and reduce SP more significantly*.

## 4. Causality and Spatial Relationship Test

### 4.1. Stationarity Test and Causation Test

Compared with previous studies, this paper presents an important development, i.e., constructing an analytical framework for the interactions between SP and EG and the two-way promotion effects of GTI on SP and EG. The panel’s simultaneous equations were used to investigate the complex relationship of GTI, SP and EG. Theoretical mechanism analysis also shows a causal interaction between SP and EG. Therefore, based on panel vector autoregression (PVAR), this paper used the Granger causal relationship test to analyze the relationship between SP and EG. Whether GTI can impact SP and EG simultaneously was also determined to provide test support for designing the panel simultaneous equation in this paper. Since PVAR estimation requires the analysis of data relationships over a long period, this paper examined the relationships between variables using panel data of 30 Chinese provinces and regions (Tibet, Hong Kong, Macao and Taiwan are excluded) from 1998 to 2020. Specifically, provincial and regional GTI levels were measured by the number of green patents of each province and region in different years, which were obtained manually from the patent search platform of the State Intellectual Property Office (SIPO) [24]. SP data are represented by raster data published by NASA’s M2TMNXAER (version 5.12.4) [5]. The data were based on the annual average of global PM_2.5_ concentrations by satellite monitoring (SP). In addition to the traditional GDP per capita at constant regional prices (EG), this paper also used nighttime light data (NTL) to measure local EG [29].

In this paper, the time series based on the provincial level covered a long period. The unit root problem may exist in the correlation series, leading to the “pseudo regression” problem in the estimation results. In order to ensure the effectiveness of the provincial-level estimation results, it is usually necessary to conduct a stationarity analysis on the relevant series data, i.e., to determine whether the data process of GTI, SP, EG and NTL is stable through the unit root test. Moreover, the stationary series is also a prerequisite for the Granger causality test. Therefore, the panel stationarity test was conducted on related sequence data before conducting the panel Granger causality test. LLC tests of the homogeneous panel hypothesis and AD-Fisher tests of the heterogeneous panel hypothesis were used to investigate robustness. The specific estimation results are shown in Table 1. All variables rejected the existence of unit root at 1% (Table 1), i.e., these four variables can pass the stationarity test at the 1% level. It can be considered that all relevant variables at the provincial level are stationary series.

Table 2 shows the Granger causality test results of the three main variables. Assumed ordinal numbers 1–4 indicate the test results of EG expressed by per capita GDP. Assumed ordinal numbers 5–8 indicate the test results of EG expressed by nighttime light brightness. In assumed ordinal numbers 2, 6, 3 and 7, the *p* values of Wald’s test were 0.084, 0.021, 0.040 and 0.029, respectively. Thus, the Wald test rejected the null hypothesis that EG and NTL do not Granger-cause SP and that SP does not Granger-cause EG and NTL at *p* ≤ 10%. This also proves a two-way causality interaction between regional EG and SP. In assumed ordinal numbers 1, 5, 4 and 8, the *p* values of the Wald test were 0.014, 0.000, 0.069 and 0.000, respectively. This indicates that the Wald test rejected the null hypothesis that GTI does not Granger-cause SP and that GTI does not Granger-cause EG and NTL at *p* ≤ 10%. This also proves that GTI has a two-way impact on regional SP and EG. According to the Granger causality test results, it is more scientific to construct a joint equation of the two-way interaction between EG and SP. The win–win analysis idea of achieving regional EG and SP control through GTI is rational.

### 4.2. Spatial Relationship Test

**Figure 1:**
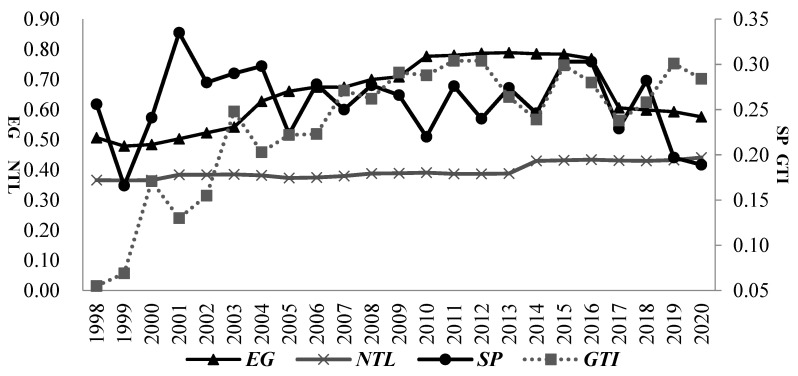
Global Moran’s I test results of the four variables.

Another significant extension of this paper is considering the spatial interaction effects of GTI, SP and EG and introducing spatial lag terms of relevant variables in the econometric estimation. Whether inter-regional spatial dependence of GTI, SP and EG exists was objectively investigated using the panel data of 30 provinces/regions in China from 1998 to 2020 as the research object. The spatial distribution of GTI, SP and EG (or NTL) in these 30 provinces/regions during the study period was also investigated using the global Moran index. The Moran’s I test results are shown in Figure 1. The left ordinate axis shows the Moran index coefficient of EG and NTL, and the right ordinate axis shows the Moran index coefficient of SP and GTI. During 1998–2020, the Moran index of EG (or NTL) was above zero (*p* < 0.1), indicating a significant spatial positive correlation between inter-regional EG. The Moran index of SP during 1998–2020 (except for 1999–2003 and 2019–2020) was more significant than zero (*p* < 0.1), indicating that the positive spatial correlation of SP was also relatively strong. The Moran index of GTI during 1998–2002 was positive (*p* > 0.1). This may be due to the relatively low GTI level in this period, and inter-regional technological exchanges were relatively limited. However, the Moran index of GTI during 2003–2016 was above zero (*p* < 0.1), indicating a relatively strong positive spatial correlation of GTI during the main research period. Comparing the Moran index coefficient values of GTI and SP shows that the spatial distribution level and changing trend of the two variables were consistent from 2013 to 2018. This may be because, in the context of the central government’s increasing attention to SP, serious regional SP drove local GTI, thus causing significant spatial dependence between GTI and SP [30].

The analysis of Moran’s I test results shows that it is reasonable to introduce spatial lag terms of variables (e.g., GTI, SP and EG) into the econometric estimation and use the spatial panel simultaneous equation for estimation.

## 5. Study Design

### 5.1. Econometric Models and Methods

Due to the inter-regional interdependence, EG in one region can significantly improve EG in neighboring regions, i.e., there exist spatial spillover effects of inter-regional EG [22]. Objective (natural conditions) and anthropogenic factors (socio-economic activities) induce a significant spatial correlation effect of inter-regional SP [5]. Inter-regional green technology transfer and agglomeration activities cause notable spatial spillover effects of GTI [23]. Moran’s I test results also confirm the above findings. Thus, we analyzed the impact of GTI is essential for SP control and EG from a spatial correlation perspective. Based on the research framework of [31], GTI, SP and EG were incorporated into a unified analysis framework. A spatial panel simultaneous equation model was established:

(1) $$SP_{it}=\alpha_{1}GTI_{it}+\alpha_{2}WGTI_{it}+\alpha_{3}EG_{it}+\alpha_{4}EG^{2}+\alpha_{5}WSP_{it}+\alpha_{6}Z_{it}+\theta_{i}+\varphi_{t}+\mu_{1it}$$

(2) $$\mu_{1it}=\rho_{1}\sum_{j=1}^{n}w_{ij}\mu_{1it}+\varepsilon_{1it}$$

(3) $$EG_{it}=\beta_{1}GTI_{it}+\beta_{2}WGTI_{it}+\beta_{3}SP_{it}+\beta_{4}WEG+\beta_{5}X_{it}+\psi_{i}+\pi_{t}+\mu_{2it}$$

(4) $$\mu_{2it}=\rho_{2}\sum_{j=1}^{n}w_{ij}\mu_{2it}+\varepsilon_{2it}$$

(5) $$\Phi =Var\left(\begin{array}{l} \varepsilon_{1it}\\ \varepsilon_{2it} \end{array}\right)=E\left(\begin{array}{cc} \varepsilon_{{}_{1it}}^{2} & \varepsilon_{{}_{1it}}\varepsilon_{{}_{2it}}\\ \varepsilon_{{}_{2it}}\varepsilon_{{}_{1it}} & \varepsilon_{{}_{2it}}^{2} \end{array}\right)==\left(\begin{array}{cc} \sigma_{11} & \sigma_{12}\\ \sigma_{21} & \sigma_{22} \end{array}\right)$$

where i is the region; t is the year; WGTI, WEG and WSP represent the spatial lag terms of local GTI, EG and SP, respectively; W is a N×N spatial weight matrix, including geographic, economic and mixed weights. Geographic weight matrix $W_{d}=1/d_{\mathrm{ab}}^{2},\;a\neq b$; otherwise, 0. Economic weights $W_{e}=1/|gdp_{a}-gdp_{b}|,\;a\neq b$; otherwise, 0. Mixed spatial weight matrix $W_{m}=W_{d}\times W_{e}$. X and Z represent exogenous control variables for the equations of EG and SP, respectively. $\psi_{i}$ and $\pi_{t}$, $\theta_{i}$ and $\phi_{t}$ refer to individual and temporal effects, respectively. $\varepsilon_{it}$ and $\mu_{it}$ are stochastic error terms.

Due to the interaction between GTI and SP, there is an endogenous problem between variables and error terms. Spatial panel joint cubic equation estimates can suffer from inconsistent or invalid estimations [31]. To overcome this problem, the exogenous control variables in the model were used as instrumental variables (IV) to obtain the corresponding predicted values. The predictors and exogenous control variables were used to regress the explanatory variables and calculate the residuals $\hat{\varepsilon }$. Next, the residual $\hat{\varepsilon }$, and the error terms ρ and Φ were estimated using the Generalized Method of Moments to get consistent estimators $\hat{\rho }$ and $\hat{\Phi }$. Then, the Cochran-Orcutt transformation was used to obtain the model equation excluding ρ and Φ. All explanatory variables obtained by the Cochran-Orcutt transformation were used to regress IV to obtain corresponding predicted values. The GLS method was used to estimate the predicted values of explanatory variables and then obtain a consistent and effective estimate of impact coefficients.

### 5.2. Variables and Data Description

A total of 278 Chinese cities during 2008–2020 were taken as the research subjects. PM_2.5_ data used in this paper were obtained from NASA’s M2TMNXAER (version 5.12.4), with a resolution of 0.625° × 0.5°. They were based on the satellite-monitored annual average global PM_2.5_ concentration. The secondary meteorological proportion of prefectural air quality was obtained from the China Environmental Yearbook, China Urban Statistical Yearbook and the data center of the Ministry of Ecology and Environment and was compiled manually by the authors. GTI data were obtained from the SIPO by setting the patent type, classification code and unit address according to the International Patent Classification (IPC) codes of green patents recognized by the World Intellectual Property Organization (WIPO). The real GDP per capita data in EG was obtained from the China Urban Statistical Yearbook. The prolonged artificial nighttime-light dataset of China from 1984 to 2020 by Zhang et al. [32] in 2021 was used, with a resolution of 30 arc seconds. This dataset was calculated using the NTL convolution long- and short-term memory neural network (ConvLSTM) released by the National Tibetan Plateau Data Center (http://data.tpdc.ac.cn/ (accessed on 21 October 2021)). Other data were derived from the China Urban Statistical Yearbook and the China Environment Statistics Annual Report. Table 3 shows the definition and statistical descriptions of the main variables.

The explained variables include SP and EG. As a state of air pollution, SP is a general expression of the content of various suspended particulate matter in the atmosphere exceeding the standard. PM_2.5_ is widely believed to cause SP and undermine air quality [33]. In addition, the air quality index (AQI) from the data center of the Ministry of Environmental Protection and the National Climate Center includes various pollutants (PM_2.5_, PM_10_ and SO_2_) and can better reflect China’s air pollution. Thus, AQI can be used to measure SP for robustness tests. Therefore, based on the study of Ma et al. (2019) [5], each city’s annual average PM2.5 concentration and AQI were used to measure SP. 

An important indicator to evaluate the national or regional EG level is GDP per capita. Official data GDP per capita were used to measure local EG levels. In addition, there may be false reporting or fraud in China’s official data. Thus, this paper referred to the practices of Du et al. (2021) [34] and used NTL data to measure EG for the robustness test.

GTI was the most important explanatory variable quantity in this paper. According to the study of Qi et al. (2022) [24], the number of regional patent applications is a good measure of local innovation. Therefore, according to the IPC codes of green patents recognized by the WIPO, the number of green patent applications was used to measure the regional GTI level. In the robustness test, based on the classification of the China Green Patent Statistical Report published by the Department of Planning and Development of the SIPO, the green patent number of different cities in different years was manually obtained on the platform search patent of the SIPO to measure the GTI level (GT).

Control variables that affect SP are as follows: ① EG level and its square term (EG and EG^2^, NTL and NTL^2^): GDP per capita and squared term of GDP per capita, as well as the nighttime light data and its square term, were used to verify whether there is an EKC in China, respectively [5]. ② Industrial structure (S): China’s traditional industrial development mode is dominated by heavy industry, which may bring greater environmental pressure and aggravate SP. Therefore, industrial development has an important impact on SP [9]. The share of the secondary sector value added to the local GDP of each region was used to represent S [10]. ③ Population density (DEN): the increase in DEN can cause urban energy consumption, traffic congestion and other problems. Therefore, it is necessary to investigate the impact of DEN on SP [21]. The ratio of the total population to the regional administrative area at the end of the year was used to represent DEN [10]. ④ Human capital (H): existing studies have found that the human capital level is closely related to local governments’ environmental concerns. In regions with higher human capital levels, people may pay more attention to the environment, which will help to strengthen local SP control [21]. The number of years of education per capita above secondary education in each region was used to represent H. ⑤ SP levels in neighboring regions (WSP): SP exhibits spatial diffusion characteristics. Thus, the spatial lag term WSP was added to the SP equation to study the influence of SP in neighboring regions on SP in the study region and then verify the agglomeration effect in the spatial distribution of SP in China [5]. ⑥ GTI levels in neighboring regions: as a form of environmental protection technology progress, GTI also has the spatial spillover effect of traditional technology [24]. Therefore, to examine the spillover effects of green technologies, the spatial lag term WGTI was added to the SP equation to measure the influence of GTI in neighboring regions on SP in the study region.

Control variables that affect EG are as follows: ① Human capital (H): an increase in the number of workers is an important driver of EG. High-quality labor (human capital) is an important guarantee for EG. Therefore, the influence of human capital on EG cannot be ignored. The number of years of education per capita above secondary education in each region was used to represent H [24]. ② Industrial structure (S): optimizing industrial structure has an important influence on high-quality economic development. Therefore, by examining the influence of the proportion of the secondary industry on EG and SP in the model, we can analyze the optimization degree and cleanliness degree of China’s current industrial structure. The share of the secondary sector value added to the local GDP of each region was used to represent S [10]. ③ Investment is one of the important driving forces to promote China’s EG. The influence of fixed asset investment on EG cannot be ignored [35]. The rate of investment in physical capital (MH) was measured by the share of fixed asset investment in regional GDP. ④ The invisible hand of the government plays an important role in EG. The degree of government intervention affects the direction of the economy. Therefore, it is necessary to examine the influence of government intervention on EG. The scale of government spending (SPE) was measured by the share of government fiscal expenditure in regional GDP [24]. ⑤ Existing studies have found that urbanization drives EG. The urbanization process can promote the development of production structures from agriculture to secondary and tertiary industries, thus facilitating EG [36]. City size (URBAN) was measured by the built-up areas of regional cities. ⑥ EG in neighboring areas (WEG): the spatial lag term WEG was added to the EG equation to study the influence of EG in neighboring regions on the SP level in the study region. Then, the agglomeration effect of inter-regional EG in China can be verified. ⑦ GTI levels in neighboring regions: the spatial lag term WGII was added to the SP equation to measure the impact of GTI in neighboring regions on the EG in the study region.

## 6. Empirical Analysis Results and Discussion

### 6.1. Impact of GTI on SP and EG under Spatial Correlation

There are mutual causal interactions between SP and EG. Significant spatial correlation effects also exist in GTI, SP and EG [5,22]. Thus, the General Spatial Three-Stage Least Squares (GS3SLS) method was used to estimate the coupled panel equations.

Table 4 shows the effects of GTI on SP and EG under spatial weight matrix estimation using geographic, economic and mixed weights, respectively. The estimation results of different types of weights were similar. Thus, this paper mainly analyzed the estimation results of mixed weights. (1) Impact of GTI on SP. The impact coefficient of local GTI on local SP was significantly negative. This indicates that the transformation and upgrading of production technologies and industrial structure adjustment due to GTI development had effectively reduced local SP. (2) Impact of GTI on EG. The impact coefficients of local GTI on EG were significantly positive, meaning that GTI can improve production efficiency and product quality to significantly promote local EG [12]. (3) Impact of EG on SP. The impact coefficient of EG on SP in the short term was significantly negative. This indicates that China has achieved some success in green development and SP control due to rapid EG, continuous optimization and upgrading of regional industrial structures, improved environmental technical standards and intensified environmental governance [37]. The above analysis shows that GTI can reduce SP directly, and indirectly by promoting regional EG, verifying the existence of Hypothesis 1 to a certain extent.

(4) Impact of SP on EG. The impact coefficient of local SP on EG in Table 4 was significantly positive, and SP control can curb EG to a certain extent. This is because SP is an undesired output of industrialization, urbanization and infrastructure construction in the context of the current crude development mode that has not been fully transformed into an intensive mode. Similarly, when the intensity of SP control increases and the SP level decreases, some production resources can be crowded out. Moreover, SP control requires large investments from the government and society. This can increase the burden on EG and thus inhibit EG to some extent [3,19]. Therefore, the analysis in (1), (2) and (4) shows that although GTI can directly promote EG, it can indirectly inhibit EG while mitigating SP to some extent. This verified the existence of Hypothesis 2.

(5) In terms of the spatial correlation impact of GTI, the impact of neighboring GTI (WGII) on SP was significantly negative, indicating that improving GTI in neighboring regions can reduce local SP. This is because, with the effective improvement of GTI in neighboring regions, local SP can also be effectively reduced through spatial correlation spillover. (6) Although the impact of neighboring GTI (WGII) on EG was positive, it did not pass the significance test statistically, indicating the insignificant role of GTI in neighboring regions in promoting local EG. GTI in neighboring regions may promote local EG through inter-regional technology spillover. However, the inter-regional technological cooperation exchange mechanism has been imperfect for a long duration, leading to a low quality of high-quality technology transfer [29]. The industrial structure adjustment and upgrading due to GTI in neighboring regions may cause local industrial upgrading through industrial spatial linkage and result in the limited promotion of local EG by green technology spillover from neighboring regions.

Among the other variables affecting SP, EG^2^ was positive for SP. EG was significantly negative for SP. Thus, there was a U-shaped relationship between EG and SP, consistent with the results of Ma et al. (2019) [5]. This is attributed to the relatively strong intensity of resource consumption and the environment’s carrying capacity in the early EG stage. However, when the EG level reaches a certain level, known as the solidified EG mode, increased energy consumption and industrial structure adjustment at the expense of others will put increasing pressure on SP from EG [6]. S was significantly positive; namely, the higher the proportion of industry-led secondary production, the more serious SP is. This is because a large percentage of heavy industrialization in the industrial structure, excessive consumption and low utilization of energy and resources will lead to an increase in pollutant emissions and aggravate SP [6]. DEN was positive. This may be because the increase in energy consumption of transportation and housing caused by the larger urban population density can aggravate SP [5]. This also shows that the current population density has not effectively exerted the intensification effect of population agglomeration. The resource utilization efficiency is too low. Thus, it is difficult to effectively reduce pollution emissions [38]. H was significantly negative, indicating that regional human capital accumulation can enhance existing regional environmental regulations and reduce the consumption of polluting resources [37]. The human capital level is also important to improve GTI [39], which is conducive to reducing SP. Although FDI was positive, it was not statistically significant, indicating that the pollution shelter hypothesis of SP still exists in China to some extent. However, with the increasing environmental regulation standards of local government after the 18th Party Congress, the exacerbating effect of FDI on regional SP was insignificant. In addition, the impact coefficient of adjacent SP (WSP) on local SP was significantly positive, indicating that SP, as regional air pollution, is affected by factors such as inter-regional industrial linkages, air flow and spatial proximity. There are significant spatial agglomeration characteristics [5].

Among the other variables affecting EG, H was significantly positive. This indicates that in the era of knowledge economy, EG is increasingly supported by connotative resource input, and the increase of human capital input can effectively promote EG. MH was significantly negative. Due to the current local pressure financial incentives, a large amount of capital flows to the real estate industry, inducing a single fixed asset investment structure. This is unfavorable to sustainable EG. S was significantly positive, indicating that industrial-based EG is essential for stable regional EG. SPE was significantly negative, indicating that governmental participation in the local economy negatively impacts EG. Excessive government intervention in the local economy can lead to unsound market EG and is not beneficial to long-term EG [19]. URBAN was significantly positive because increasing city size can lead to increased infrastructure investment and EG [36]. In addition, the impact coefficient of neighboring EG (WEG) on local EG was significantly positive, indicating a significant spatial agglomeration effect on inter-regional EG due to the correlation between industrial linkages, resource factors and human flows [22].

### 6.2. Impact of Different Types of GTI on SP and EG

Table 5 shows the measured estimates of different types of GTI on SP and EG. The impact coefficients of control variables of the SP and EG equations are similar to those in Table 4 and are not duplicated. The left side of Table 5 shows the impact of GII on SP and EG measured by green invention and innovation patents. Specifically, the impact coefficients of GII on SP and EG were −0.448 and 0.140, passing the significance test at 1% and 5%, respectively. The right side of Table 5 shows that the impact coefficients of GUMI on SP and EG were −0.081 and 0.090, passing the significance test at 5% and 10%, respectively. This means that GII and GUMI can significantly contribute to EG and reduce SP. In addition, the influence of WGII on SP (−0.513) and EG (0.092) in neighboring regions was also greater than that of WGUMI (−0.002 and 0.074). The results passed the significance test, indicating that both WGII and WGUMI can significantly promote EG and reduce SP. However, these results also indicate that, compared with GUMI (WGUMI), GII (WGII) has a stronger direct inhibition effect on SP and an indirect inhibition effect on SP through EG, verifying the existence of Hypothesis 3 to some extent. Because the patents of GII have higher innovation and higher green technology content [27], they can effectively promote the clean transformation of production processes and the optimization and upgrading of industrial structures to reduce SP. Furthermore, GII, as a substantive innovation, can effectively enhance the market value of enterprises, realize green innovation-led development and promote EG [26].

### 6.3. Analysis of the Comprehensive Impact of GTI on SP and EG

In the context that China’s EG mode has not been fully and effectively transformed and upgraded, the contradiction between SP and EG is still prominent in most regions. GTI can facilitate resolving this contradiction. Based on the estimation results in Table 4 and Table 5, this paper estimated the comprehensive impact of GTI on local SP and EG. It is found that green technology effectively reduced SP through both direct and indirect effects on EG, verifying the existence of Hypothesis 1. GTI directly increased EG but inhibited regional EG to a certain extent by reducing SP. The comprehensive impact shows that GTI can effectively promote regional EG, verifying the existence of Hypothesis 2. The comparison of different types of GTI in Table 6 reveals that the positive impact of GII on promoting EG and suppressing SP was significantly greater than that of GUMI. This verified the existence of Hypothesis 3. A comprehensive analysis of the impact of GTI on SP and EG reveals that regardless of the GTI variables, GTI can effectively achieve the win–win situation of SP reduction and EG.

### 6.4. Robustness Test

The research conclusions based on Table 4 systematically verified Hypotheses 1 and 2. However, in the previous estimate, some selected indicators may be inaccurately measured for various reasons. Thus, their robustness was tested through a series of variable substitutions. Firstly, EG was measured by regional per capita income (EG), which usually does not fully and effectively reflect regional EG because of crude price deflators and flawed statistical data reporting mechanisms. In recent years, this issue has been effectively addressed using regional stable NTL data [5]. Therefore, in Column 1 of Table 7, per capita income (EG) data were replaced by NTL data from 278 municipalities during 2008–2020 to examine the impact of GTI on regional EG and SP. Secondly, the PM_2.5_ concentration (the SP index) in the previous study was not published by official Chinese sources and may deviate from the actual situation. In Column 2 of Table 7, the degree of SP was measured by determining the proportion of days with air quality reaching or better than Grade II (AQI) in 278 cities from 2016 to 2020 (considering the availability of data) and testing the robustness. This index was obtained from the total concentration of multiple air pollutants and reflected a comprehensive evaluation of the air pollution status throughout the year. It provides a more effective measure of regional air pollution status. A higher index indicates higher air quality and a lower SP level. Finally, the GTI indicators in the above study were based on green patent data from the green patent list provided by WIPO. The data may deviate from China’s actual green patent standards and development. Therefore, In Column 3 of Table 7, based on the green patent data collected by the SIPO as the keywords, GT was measured, and robustness testing was conducted. These data can more effectively reflect the GTI level in China.

In this paper, GS3SLS estimation was used to econometrically estimate the simultaneous equation after the above data replacement. The robustness test results are shown in Table 7. After replacing the relevant variables, GTI can reduce SP directly, or indirectly by promoting inter-regional EG in all equations. GTI can directly promote EG, but adversely affect EG by reducing inter-regional SP. Therefore, it can be concluded that the spatial correlation estimation results of GTI on SP and EG in this paper were robust and reliable, further verifying the existence of Hypotheses 1 and 2.

Similarly, the proportion of days with air quality no lower than Grade II (AQI) in each city was used as an inverse measure of the SP level. This paper further tested the robustness of the impact of different types of GTI on AQI and EG (Table 8). The results show that the impact coefficients of GII on AQI and EG were 0.005 and 0.142, respectively, passing the significance test at 1%. However, the impact coefficients of GUMI on AQI and EG were 0.003 and 0.079, passing the significance test at 5% and 1%, respectively. Compared with GUMI, GII had a more prominent impact on improving AQI and promoting EG, verifying the existence of Hypothesis 3.

## 7. Conclusions

In this paper, the data of 278 cities in China during 2008–2020 were analyzed. The dynamic spatial panel simultaneous equation estimation method was used to systematically investigate the impact of GTI on SP and EG. The main conclusions are as follows: (1) GTI can reduce SP directly, and indirectly by promoting inter-regional EG. GTI can directly promote EG but indirectly inhibit EG by alleviating inter-regional SP. GTI in neighboring regions can also facilitate local SP. There are significant spatial agglomeration characteristics of inter-regional SP. EG has a significant positive spatial correlation. (2) Compared with GUMI, GII has a more significant effect on reducing SP and promoting EG. (3) The analysis of the comprehensive impact of GTI on SP and EG shows that GTI can generally achieve the coordinated development of SP reduction and stable EG regardless of GTI types.

## 8. Implications

This study aimed to clarify the balanced role of GTI on SP and EG. Based on the above main findings, the following policy insights were obtained:

(1) GTI is an effective means to coordinate SP reduction and stabilize EG. We should delineate the responsibilities of government and enterprises in GTI. The report of the 19th Party Congress has proposed that green development should be effectively achieved through market-oriented GTI construction. The government should formulate scientific and rational environmental regulatory policies, strengthen R&D support for GTI and effectively guide social capital to enter green industries. Enterprises should contribute from the following aspects: establish the awareness of innovation and sustainable development; actively recruit and cultivate green innovation talents; promote the effective development of GTI activities; continuously improve the conversion rate of green technology achievements. Thus, the coordinated development of improved EG quality and efficiency and reduced SP and emissions can be achieved.
(2) Fighting the Blue Sky Defense Battle is important to continuously deepen the battle against pollution. Local governments should actively implement the concept that clean water and green mountains are valuable assets. They should also continuously improve the green performance evaluation system, scientifically assess the bearing capacity of environmental resources and strictly adhere to the ecological protection red line. In the current stage of overcoming difficulties and deepening SP control, the government should continue to strengthen the environmental regulation of key polluting enterprises and the accountability and supervision of environmental protection departments. The government should also actively enhance public awareness of environmental protection and create an excellent social atmosphere for everyone to participate in smog control. In addition, smog is a composite pollutant with spatial transmission and negative externality. Thus, local governments should actively implement the three-year action plan to win the Blue Sky Defense Battle, build a cross-regional joint prevention and control mechanism and coordinate regional smog control.
(3) GTI and high-quality coordinated regional economic development should be promoted. China is still in an important stage of shifting its EG rate, adjusting its industrial structure and transforming the development mode. We should abandon the EG mode that emphasizes the economy and underplays environmental protection. We should also adopt the new concept of green, low-carbon and circular development, vigorously promote green technological progress, stimulate the transformation and upgrading of industrial structure and promote high-quality economic development. We must actively promote cleaner production and end-of-line treatment technologies, achieve both source prevention and end-of-line treatment, consolidate and improve pollution prevention and control achievements, reduce environmental treatment costs and economic burden and achieve sustainable EG. In addition, the government should coordinate the planning of the development of economic zones and the construction of urban agglomerations, break inter-regional market barriers and accelerate the flow of factors and inter-regional technological exchanges and cooperation. Furthermore, the government should constantly promote the transformation of its functions, improve resource allocation efficiency and promote regional coordinated economic development.

## Figures and Tables

**Table 1:** Panel stationarity test of variables.

Variables	Inspection Form (c,t,l)	LLC Test		ADF-Fisher Test	
		Statistic	*p*-Value	Statistic	*p*-Value
GTI	(c,t,1)	−13.7964	0.0000	88.9574	0.0090
SP	(c,t,1)	−10.9962	0.0000	156.7073	0.0000
EG	(c,t,1)	−9.8766	0.0004	104.0423	0.0004
NTL	(c,t,1)	−11.5741	0.0000	138.1501	0.0000

Note: c, t and l represent the order with the constant term, trend term and lag, respectively.

**Table 2:** Granger causality test of variables.

Assumed Ordinal Number	Null Hypothesis	Wald	*p*	Test Results
1	GTI does not Granger-cause SP	14.337	0.014	refuse
2	EG does not Granger-cause SP	8.204	0.084	refuse
3	SP does not Granger-cause EG	10.031	0.040	refuse
4	GTI does not Granger-cause EG	8.707	0.069	refuse
5	GTI does not Granger-cause SP	29.016	0.000	refuse
6	NTL does not Granger-cause SP	13.23	0.021	refuse
7	SP does not Granger-cause NTL	12.446	0.029	refuse
8	GTI does not Granger-cause NTL	25.183	0.000	refuse

**Table 3:** Granger causality test results of the variables.

Variables	Definition	Number of Samples	Mean	Standard Deviation	Minimum	Maximum
SP	Smog pollution (μg/m^3^)	3614	45.06	15.34	13.45	98.53
EG	Per capita income (million)	3614	4.41	3.03	0.10	11.55
S	Industrial structure (%)	3614	47.70	10.85	10.93	90.97
H	Human capital (year)	3614	2.03	0.487	0.07	3.81
MH	Rate of investment in physical capital (%)	3614	73.68	17.62	3.21	290.28
SPE	Scale of government spending (%)	3614	15.27	8.06	4.18	153.62
URBAN	City size (100 km^2^)	3614	1.35	1.88	0.07	33.71
DEN	Population density (Person/km^2^)	3614	438.019	338.895	4.82	2713.02
GT	Green patent—keywords (piece)	3614	17.06	32.55	0.00	607.29
GTI	Green patent—classification number (piece)	3614	60.48	198.26	0	3657
GII	Green invention patent (piece)	3614	31.69	116.80	0	2643
GUMI	Green utility model patent (piece)	3614	28.78	82.67	0	1248
NTL	Nighttime light data (-)	3614	7.32	7.92	0.17	56.61
NTL^2^	Squared term of nighttime light data (-)	3614	45.06	15.34	13.45	98.53
AQI	The proportion of air quality above Grade II (-)	1390	0.776	0.156	0.31	1

**Table 4:** Effects of GTI on SP and EG.

Geographic Weights				Economic Weights				Mixed Weights			
SP		EG		SP		EG		SP		EG	
C	23.98 ***	C	−6.812 ***	C	12.47 ***	C	2.884 ***	C	12.72 ***	C	−2.745 ***
	(2.29)		(−10.38)		(8.72)		(10.28)		(7.20)		(−6.81)
EG	−1.701 ***	SP	0.291 ***	EG	−1.788 **	SP	0.139 ***	EG	−1.114 ***	SP	0.241 ***
	(−4.50)		(26.62)		(−2.37)		(2.95)		(−2.12)		(28.41)
EG^2^	0.067 ***	H	2.296 ***	EG^2^	0.035 ***	H	1.333 ***	EG^2^	0.013 ***	H	1.934 ***
	(2.93)		(19.77)		(7.54)		(14.13)		(4.10)		(18.43)
S	0.416 ***	MH	−0.188 ***	S	0.511 ***	MH	−0.520 ***	S	0.464 ***	MH	−0.249 ***
	(26.69)		(−5.49)		(21.89)		(−5.85)		(25.45)		(−7.08)
GTI	−0.114 ***	GTI	0.043 ***	GTI	−0.258 ***	GTI	0.062 ***	GTI	−0.176 ***	GTI	0.054 ***
	(−12.54)		(15.40)		(−20.16)		(24.72)		(−17.29)		(20.49)
WGTI	−0.178 **	WGTI	0.032	WGTI	−0.002 *	WGTI	0.001	WGTI	−0.257 ***	WGTI	0.052
	(−2.52)		(1.38)		(−1.65)		(1.33)		(−5.68)		(4.14)
DEN	0.003 ***	S	0.131 ***	DEN	0.011 ***	S	0.109 ***	DEN	0.004 ***	S	0.129 ***
	(5.93)		(22.65)		(17.07)		(22.90)		(7.44)		(24.68)
H	−6.767 ***	SPE	−0.037 *	H	−3.829 ***	SPE	−0.145	H	−6.024 ***	SPE	−0.086 *
	(−18.07)		(−1.72)		(−6.79)		(−0.95)		(−13.13)		(−1.86)
FDI	0.666	URBAN	0.003 *	FDI	0.484	URBAN	0.003	FDI	0.349	URBAN	0.004 *
	(0.19)		(1.92)		(0.07)		(0.01)		(0.08)		(1.72)
WSP	0.914 ***	WEG	0.620 ***	WSP	0.002 *	WEG	0.001 *	WSP	0.633 ***	WEG	0.307 ***
	(22.30)		(5.77)		(1.83)		(1.82)		(19.21)		(5.14)
R^2^	0.79	R^2^	0.25	R^2^	0.89	R^2^	0.76	R^2^	0.87	R^2^	0.58

Note: ( ) indicates the *t*-test value of the parameter; ***, ** and * denote *t*-value significant at the statistical level of 1%, 5% and 10%, respectively.

**Table 5:** Impacts of different types of GTI on SP and EG.

Green Invention and Innovation (GII)				Green Utility Model Innovation (GUMI)			
SP		EG		SP		EG	
C	11.88 ***	C	−2.506 ***	C	13.466 ***	C	−2.920 ***
	(6.59)		(−6.37)		(19.93)		(−7.12)
EG	−1.334 **	SP	0.229 ***	EG	−1.754 **	SP	0.248 ***
	(−2.51)		(27.66)		(−2.44)		(28.61)
EG^2^	0.122 ***	H	1.843 ***	EG^2^	0.148 ***	H	2.011 ***
	(3.76)		(18.18)		(4.66)		(18.71)
S	0.466 ***	MH	−0.237 ***	S	0.453 ***	MH	−0.278 *
	(25.56)		(−6.91)		(24.82)		(−1.75)
GII	−0.448 ***	GII	0.140 **	GUMI	−0.081 **	GUMI	0.090 *
	(−7.50)		(2.32)		(−2.28)		(1.83)
WGII	−0.513 ***	WGII	0.092 ***	WGUMI	−0.002 **	WGUMI	0.074
	(−2.92)		(3.27)		(−2.19)		(1.17)
DEN	0.004 ***	S	0.125 ***	DEN	0.004 ***	S	0.130 ***
	(7.93)		(24.89)		(7.45)		(24.11)
H	−5.973 ***	SPE	−0.093	H	−6.019 ***	SPE	−0.079 ***
	(12.97)		(−0.68)		(−13.11)		(−2.57)
FDI	0.586	URBAN	0.005 ***	FDI	1.205	URBAN	0.003 **
	(0.13)		(7.34)		(0.27)		(2.21)
WSP	0.618 ***	WEG	0.302 ***	WSP	0.648 ***	WEG	0.325 ***
	(18.53)		(5.00)		(19.93)		(5.54)
R^2^	0.87	R2	0.61	R^2^	0.87	R^2^	0.56

Note: ***, ** and * denote *t*-value significant at the statistical level of 1%, 5% and 10%, respectively.

**Table 6:** Differences in the impact of GTI on SP and EG.

Direction of Action	Pathway	Green Technology Innovation (GTI)	Green Invention and Innovation (GII)	Green Utility Model Innovation (GUMI)
Smog pollution	Direct impact	−0.176	−0.448	−0.081
	Economic growth—smog pollution pathways	−0.054	−0.036	−0.040
	Combined impact	−0.230	−0.484	−0.121
Economic growth	Direct impact	0.054	0.140	0.090
	Smog pollution—economic growth pathways	−0.042	−0.102	−0.020
	Combined impact	0.012	0.038	0.070
Can it be balanced?		Yes	Yes	Yes

Note: The coefficients were calculated based on Table 3 and Table 4. Table 3 uses the mixed weight estimation results, and the sample mean value of 44,110 CNY was used as the per capita income (EG).

**Table 7:** Robustness test results of the impact of GTI on SP and EG.

Economic Growth (NTL)				Smog Pollution (AQI)				Green Technology Innovation (GT)			
SP		NTL		AQI		EG		SP		EG	
C	3.267 **	C	−2.333 ***	C	0.769 ***	C	0.102 ***	C	4.035 ***	C	−0.562 ***
	(2.00)		(−2.85)		(13.97)		(3.48)		(3.76)		(−2.90)
NTL	−0.308 *	SP	0.456 ***	EG	0.026 **	AQI	−16.62 ***	EG	−0.079 ***	SP	0.011 **
	(−1.78)		(9.75)		(1.99)		(−5.28)		(−9.28)		(1.93)
NTL^2^	0.063 **	H	2.612 ***	EG^2^	−0.003 ***	H	2.448 ***	EG^2^	0.027 ***	H	0.943 ***
	(2.05)		(10.36)		(−4.74)		(15.61)		(4.67)		(5.57)
S	0.237 ***	MH	−0.213 ***	S	−0.004 *	MH	−0.234 ***	S	0.031 ***	MH	−0.272 ***
	(12.75)		(−4.00)		(−1.92)		(−5.55)		(3.63)		(−2.61)
GTI	−0.189 ***	GTI	0.014 ***	GTI	0.002 ***	GTI	0.054 ***	GT	−0.215 ***	GT	7.821 ***
	(17.59)		(7.94)		(3.11)		(18.70)		(−5.18)		(10.65)
WGTI	−0.470 ***	WGTI	0.003	WGTI	0.006 *	WGTI	0.017	WGT	−0.017 **	WGT	0.061
	(−9.72)		(1.51)		(1.74)		(1.40)		(−1.99)		(0.98)
DEN	0.013 ***	S	0.063 ***	DEN	−0.008	S	0.013 ***	DEN	−0.003	S	0.040 ***
	(10.95)		(4.87)		(−0.61)		(16.87)		(−0.22)		(16.28)
H	−1.019 **	SPE	0.057	H	0.049 ***	SPE	−0.096 ***	H	−1.796 ***	SPE	−0.055 ***
	(−2.09)		(1.34)		(2.52)		(−5.03)		(−4.09)		(−2.55)
FDI	2.155	URBAN	0.006 ***	FDI	−0.003	URBAN	0.034 **	FDI	0.030	URBAN	0.061 ***
	(0.35)		(2.13)		(−0.04)		(2.35)		(0.79)		(6.51)
WSP	0.816 ***	WNTL	0.013 ***	WAQI	0.059 ***	WEG	0.708 ***	WSP	0.634 ***	WEG	0.317 **
	(26.27)		(21.03)		(9.68)		(7.17)		(3.07)		(2.16)
R^2^	0.80	R2	0.55	R2	0.90	R2	0.77	R2	0.91	R2	0.75

Note: ***, ** and * denote *t*-value significant at the statistical level of 1%, 5% and 10%, respectively.

**Table 8:** Robustness test results of the impact of different types of GTI on SP and EG.

Green Invention Innovation (GII)				Green Utility Model Innovation (GUMI)			
AQI		EG		AQI		EG	
C	0.769 ***	C	9.866 ***	C	0.766 ***	C	9.956 ***
	(13.67)		(9.15)		(14.09)		(9.22)
EG	0.021 *	AQI	−16.51 ***	EG	0.032 ***	AQI	−16.01 ***
	(1.67)		(−5.33)		(2.43)		(−4.50)
EG^2^	−0.003 ***	H	2.288 ***	EG^2^	−0.003 ***	H	2.562 ***
	(−4.53)		(14.65)		(−5.01)		(16.38)
S	−0.004 ***	MH	−0.203 ***	S	−0.004 ***	MH	−0.296 ***
	(−7.17)		(−4.84)		(−6.24)		(−6.99)
GII	0.005 ***	GII	0.142 ***	GUMI	0.003 **	GUMI	0.079 ***
	(3.70)		(9.78)		(2.09)		(7.03)
WGII	0.003 *	WGII	0.060 *	WGUMI	0.003	WGUMI	0.019
	(1.89)		(1.85)		(0.39)		(0.98)
DEN	−0.008 ***	S	0.128 ***	DEN	−0.009	S	0.129 ***
	(−7.27)		(6.759)		(−1.38)		(6.39)
H	0.047 ***	SPE	−0.024 ***	H	0.046 ***	SPE	−0.027 ***
	(3.44)		(−4.90)		(3.31)		(−5.27)
FDI	−0.003	URBAN	0.061 ***	FDI	−0.009	URBAN	0.041 ***
	(−0.04)		(6.29)		(−0.11)		(5.83)
WAQI	0.601 ***	WEG	0.714 ***	WAQI	−0.588 ***	WEG	0.694 ***
	(9.65)		(6.63)		(−5.54)		(2.72)
R^2^	0.89	R2	0.79	R2	0.92	R2	0.76

Note: ***, ** and * denote *t*-value significant at the statistical level of 1%, 5% and 10%, respectively.

## Data Availability

Data sharing is not applicable to this article.

## Abbreviations

SP	Smog pollution
GTI	Green technology innovation
GII	Green invention and innovation
EKC	Environmental Kuznets Curve
R&D	Research and development
PVAR	Panel vector autoregression
NTL	Nighttime light data
AQI	Air quality index
WIPO	World Intellectual Property Organization
H	Human capital
SPE	Scale of government spending
DEN	Population density
EG	Economic growth
GUMI	Green utility model innovation
CPC	Communist Party of China
OECD	Organization for Economic Co-operation and Development
SIPO	State Intellectual Property Office
NOAA	National Oceanic and Atmospheric Administration
GS3SLS	General Spatial Three-Stage Least Squares
IPC	International Patent Classification
S	Industrial structure
MH	Rate of investment in physical capital
URBAN	City size
